# Editorial: Precision neuroimaging for MRgFUS in neurological disorders

**DOI:** 10.3389/fradi.2026.1979646

**Published:** 2026-09-15

**Authors:** Federico Bruno, Sotirios Bisdas

**Affiliations:** 1 Interventional Radiology and Neuroradiology, San Salvatore Hospital, L'Aquila, Italy; 2 University College London, London, United Kingdom

**Keywords:** MRgFUS, MRI, radiomics, thalamotomy, tremor

Magnetic resonance-guided focused ultrasound (MRgFUS) is redefining functional neurosurgery by combining incisionless thermal ablation with real-time MRI guidance. Its clinical adoption for medication-refractory essential tremor and tremor-dominant Parkinson's disease has made millimetric targeting, lesion monitoring, and post-treatment assessment immediate practical priorities. At the same time, growing interest in staged bilateral procedures and alternative circuit-based targets is increasing the anatomical and biological complexity of treatment. In this setting, neuroimaging is no longer only the navigation platform for sonication; it is becoming the connective tissue of the therapeutic pathway, from target definition and intraoperative decision-making to mechanistic interpretation, outcome assessment, and, potentially, individualized prognosis.

This Research Topic captures that transition through five complementary contributions spanning direct thalamic visualization, multimodal localization of emerging targets, longitudinal network analysis, radiomics-based outcome modeling, and quantitative assessment of staged bilateral treatment. Together, these studies position precision neuroimaging not as a single sequence or technique, but as an operational framework: one that links patient-specific anatomy to circuit organization, procedural execution, tissue response, and clinical follow-up. Their collective message is timely. As MRgFUS expands, the field will need imaging strategies that are not only more informative, but also reproducible, interpretable, and demonstrably useful across centers.

At the level of target definition, Aibar-Duran et al. address a longstanding limitation of ventral intermediate nucleus (Vim) thalamotomy: reliance on indirect atlas-based coordinates despite meaningful interindividual variation. The authors describe the use of 3-T white-matter-nulled MPRAGE imaging to visualize the Vim and neighboring structures, combined with a dual-lesion strategy intended to conform to the nucleus's oblique anterior-superior orientation. Their experience supports the feasibility of integrating direct anatomical information into routine MRgFUS planning and illustrates how target coverage may be adapted to individual anatomy. Importantly, the report does not establish superiority over conventional targeting or provide comparative clinical validation. Its value is therefore both practical and agenda-setting: it shifts the question from where an atlas predicts the target to how confidently that target and its boundaries can be identified in the patient being treated.

Precision also requires recognizing what current imaging cannot yet resolve. Rivera et al. examine the pallidothalamic region, an emerging circuit-based target for Parkinson's disease in which the relevant fibers remain difficult to visualize directly. Their mini review integrates high-resolution structural MRI, inversion-recovery and susceptibility-based contrasts, and diffusion tractography, and highlights the mammillothalamic tract as a comparatively consistent adjacent landmark. Crucially, the mammillothalamic tract is not proposed as the therapeutic target or as a fixed millimetric offset; rather, it may help orient the pallidothalamic corridor while identifying a memory-related pathway that should be preserved. This distinction exemplifies a mature use of multimodal imaging: not to claim visibility that is not yet achievable, but to reduce anatomical uncertainty through complementary information. Prospective studies must still determine whether this approach improves targeting accuracy, safety, or clinical outcomes.

Once a lesion has been placed, imaging can interrogate consequences that extend beyond the ablation site. Jiang et al. studied resting-state functional connectivity before and one year after unilateral Vim thalamotomy in 15 patients with essential tremor. Sustained improvement in the treated hand was accompanied by altered connectivity involving the Vim, primary motor cortex, dentate nucleus, and distributed motor, sensory, attentional, visual, and visuospatial regions; changes in left Vim connectivity were also associated with changes in tremor severity. These observations support a network-level interpretation of MRgFUS, in which a focal lesion is associated with remote and bilateral functional reorganization. They do not, however, establish causality or a validated predictive biomarker. Larger controlled cohorts, complemented by tractography and standardized cognitive assessment, will be needed to determine which connectivity changes mediate benefit, reflect compensation, or simply accompany recovery.

Where functional MRI looks outward from the lesion to the network, radiomics looks inward to quantify tissue patterns that conventional inspection may not capture. Innocenzi et al. analyzed 24-hour post-treatment T2-weighted MRI in 120 patients undergoing unilateral MRgFUS thalamotomy and used a machine-learning model to classify clinically relevant tremor recurrence at 12 months. Recurrence occurred in 23 patients (19%), and the internally cross-validated model achieved a balanced accuracy of 0.720 in the full cohort, with stronger performance in Parkinson's disease than in essential tremor. Texture features contributed most strongly to the model, suggesting that the early lesion phenotype may contain information related to treatment durability. The study is appropriately viewed as a pilot: its single-center design, modest event count, diagnostic subgroup differences, class imbalance, manual segmentation, and absence of external validation preclude immediate clinical deployment. Nevertheless, it offers a concrete route from routine post-procedural MRI toward testable, quantitative prognostic tools.

Paio et al. extend the precision-imaging agenda to staged bilateral thalamotomy, where reproducibility must be balanced against the risks of bilateral intervention. In their single-center cohort of 15 patients, most anatomical, procedural, and sonication parameters were comparable between the first- and second-side procedures, as were lesion volumes at 24 h and approximately one month. Second-side targeting incorporated a small anterior-dorsal refinement rather than strict mirror symmetry, and adverse events were predominantly mild and transient. Neither lesion volume nor inter-side targeting displacement distinguished patients with gait disturbance persisting at one month, while individual imaging patterns suggested that lesion geometry, extension into adjacent structures, and network effects may all matter. The study thus makes an important conceptual point: in bilateral MRgFUS, precision is not synonymous with geometric symmetry, but with cautious, imaging-informed adaptation.

Read together, the five contributions form a coherent translational arc (Aibar-Duran et al.; Rivera et al.; Jiang et al.; Innocenzi et al.; Paio et al.). Before treatment, structural and diffusion imaging can reduce uncertainty in target localization and identify structures at risk. During treatment, MRI provides thermometric feedback and supports clinically guided refinement. After treatment, lesion volumetry, radiomics, and functional connectivity can characterize tissue response, procedural consistency, and distributed network change. This continuum matters because MRgFUS outcomes cannot be reduced to a coordinate, a peak temperature, or a lesion volume in isolation. Durable benefit and acceptable risk are more likely to depend on the interaction among patient anatomy, circuit architecture, lesion geometry, biological response, and functional reserve.

The next phase of the field should therefore be defined by validation and integration. Harmonized acquisition, segmentation, tractography, and outcome definitions are needed to make results comparable across institutions. Prospective multicenter studies should combine structural, diffusion, functional, and quantitative lesion imaging with clinical and procedural variables, while testing models on genuinely external data. Patient-specific connectomics, computational modeling, radiomics, and explainable artificial intelligence could ultimately help refine target selection, anticipate benefit and adverse effects, and tailor follow-up. Their clinical value, however, will depend on transparent uncertainty, reproducibility, and evidence that imaging-informed decisions improve outcomes. This Research Topic does not present a finished solution; more usefully, it defines a credible and testable roadmap for precision neuroimaging in MRgFUS.

We thank all authors who contributed to this Research Topic for their work and perspectives, and the reviewers whose thoughtful evaluations strengthened the published articles. As MRgFUS moves toward more complex targets, staged bilateral strategies, and more individualized care, precision neuroimaging will be central to translating technical capability into durable, safe, and generalizable clinical benefit. We hope this collection will serve both as a practical resource and as a catalyst for the collaborative studies needed to realize that potential.

